# Microbial signatures of dental caries in the incarcerated elderly: a salivary microbiota study in a restricted environment

**DOI:** 10.1080/20002297.2026.2662787

**Published:** 2026-04-29

**Authors:** Teeratas Kijpornyongpan, Annop Krasaesin, Thananya Chongcharoenkit, Khanti Rattanapornsompong, Avirut Truntipakorn, Patita Bhuridej, Nadnudda Rodthongkum, Sung-Dae Cho, Thantrira Porntaveetus

**Affiliations:** a Center of Excellence in Precision Medicine and Digital Health, FutureDent Digital Center, Department of Physiology, Faculty of Dentistry, Chulalongkorn University, Bangkok, Thailand; b Master of Science Program in Geriatric Dentistry and Special Patients Care, Faculty of Dentistry, Chulalongkorn University, Bangkok, Thailand; c Bang Kwang Central Prison, Department of Corrections of Thailand, Ministry of Justice, Nonthaburi, Thailand; d Department of Community Dentistry, Faculty of Dentistry, Chulalongkorn University, Bangkok, Thailand; e Department of Chemistry, Faculty of Science, Chulalongkorn University, Bangkok, Thailand; f Department of Oral Pathology, School of Dentistry and Dental Research Institute, Seoul National University, Seoul, Republic of Korea; g Clinic of General, Special Care and Geriatric Dentistry, Center for Dental Medicine, University of Zürich, Zürich, Switzerland

**Keywords:** Dental caries, geriatric oral health, microbiome, restricted environments, 16S rRNA sequencing, beta diversity, healthcare disparity

## Abstract

**Background:**

Dental caries is driven by microbial dysbiosis and influenced by diet and lifestyle. Incarcerated populations living under regulated regimens offer a unique model to study the oral microbiota in older adults by minimising environmental confounding variables.

**Objectives:**

This study aimed to characterise the salivary microbiota of older incarcerated adults and identify bacterial taxa associated with caries status and severity.

**Design:**

Twenty-eight incarcerated men (aged ≥ 50 years) were stratified into caries-active (CA) and caries-free (CF) groups (*n* = 14 each). The salivary microbiota was profiled using 16S rRNA gene sequencing to assess diversity and differential taxonomic abundance.

**Results:**

CA subjects exhibited higher genus richness and beta-dispersion compared to CF controls. The CF group was enriched with *Haemophilus parainfluenzae*, *Aggregatibacter* sp. HMT-949 and *Riemerella* sp. HMT-322. Conversely, the CA group harboured elevated levels of *Dialister invisus*, *Megasphaera micronuciformis*, *Prevotella intermedia*, *Selenomonas sputigena*, *Capnocytophaga ochracea* and *Gemella haemolysans*. Furthermore, *G. haemolysans, Solobacterium moorei* and *Streptococcus* were positively correlated with caries severity, whereas *Veillonella rogosae* and *Streptococcus koreensis* and *Peptostreptococcus*exhibited negative correlation.

**Conclusion:**

This study elucidates salivary dysbiotic signatures in older adults within a controlled environment. The identified bacterial profiles provide biomarkers for caries risk, underscoring the need for targeted oral health surveillance and preventative strategies in institutionalised populations.

## Introduction

Dental caries remains a pervasive global health burden, disproportionatelyimpacting quality of life across the lifespan [1]. While the aetiology of childhood caries has been extensively characterised [2], the microbiological landscape of caries in the elderly remains poorly defined. This knowledge gap is critical; caries in older adults is distinct and multifactorial, driven by unique age-related complexities including gingival recession, polypharmacy-induced xerostomia and immunosenescence [3,4]. As the global population ages, elucidating the specific biological mechanisms driving geriatric caries has become a critical public health priority.

Recent advancements in high-throughput sequencing have facilitated a paradigm shift in the understanding of caries aetiology, moving beyond the identification of specific pathogens toward a more holistic characterisation of polymicrobial dysbiosis [5,6]. While classical biomarkers such as *Streptococcus mutans*, *Lactobacillus* and *Candida albicans* are well-documented [7], metagenomic data increasingly suggest that these taxa are not universally predictive, particularly in non-paediatric cohorts. The elderly oral microbiome, characterised by distinct ecological pressures, exhibits high variability and inconsistent taxonomic correlations in the limited studies available [8,9]. Consequently, the precise microbial signatures associated with active caries in older adults remain elusive, obscured by the vast heterogeneity of lifestyle and dietary factors in the general population. In addition, the oral microbiota is influenced by host genetic background, ethnicity and geographic location. Although large-scale, comprehensive studies of the oral microbiome have been conducted in global and Asian populations, data remain limited for Southeast Asian populations [10–12].

To address this heterogeneity, we leverage a unique study population: incarcerated older adults. While incarceration is clinically associated with heightened caries susceptibility due to limited care access [13,14], scientifically, it represents a restricted environment that naturally controls for extrinsic confounders. Unlike community-based studies, where diet and hygiene habits vary wildly, the incarcerated population lives under a standardised regimen. This minimises environmental noise, allowing for a clearer resolution of the biological signal driving the caries-associated microbiome.

In this study, we profiled the salivary microbiota of elderly prisoners to identify bacterial taxa specifically associated with active dental caries. Furthermore, recognising the interplay between systemic and oral health, we evaluated the influence of hypertension and smoking history as covariates. By characterising the oral ecosystem in this controlled setting, we aim to isolate specific microbial biomarkers of caries in the ageing population, distinct from environmental variability.

## Materials and methods

### Study site and population

This study was conducted at Bang Kwang Central Prison, Nonthaburi, Thailand. Recruitment and data collection took place between June and December 2024. Demographic information and medical histories for all potential participants were retrieved from the prison's electronic health record database.

All participants underwent a comprehensive clinical oral examination conducted by trained and calibrated dentists. Examinations were performed under artificial illumination using plane mouth mirrors and World Health Organisation (WHO) Community Periodontal Index (CPI) probes. Dental status was recorded in accordance with WHO criteria to assess the prevalence and severity of dental caries.

### Subject selection

Participants included male inmates aged 50 years and older. Subjects were stratified into two groups based on their caries status: the caries-active (CA) group, defined as individuals having at least two decayed teeth (DT ≥ 2) and the caries-free (CF) group, defined as individuals with no decayed teeth (DT = 0). This classification criterion is consistent with methodologies used in previous salivary microbiome studies [9,15,16].

The exclusion criteria were as follows: (1) presence of uncontrolled systemic diseases, (2) fewer than eight remaining natural teeth, (3) current smoking habits, (4) use of removable dental prostheses or fixed orthodontic appliances, and (5) refusal to provide informed consent.

A total of 28 subjects were enrolled in the study (*n* = 14 for the CA group; *n* = 14 for the CF group). The final cohort included participants with controlled systemic conditions, specifically controlled hypertension (CA: *n* = 6; CF: *n* = 6), controlled diabetes (CA: *n* = 5; CF: *n* = 3) and smoking history (CA: *n* = 6; CF: *n* = 5). We noted that subjects with a smoking history have quit smoking for at least 5 years. All subjects presented with clinical features consistent with periodontitis. Detailed demographic and clinical characteristics of the study population are presented in Table S1.

### Sample collection

Participants were required to abstain from food, beverages and oral hygiene procedures for a minimum of 2 h before collection. Samples were obtained during a morning window (08.00–11.00 am) to maintain consistency across the cohort. Unstimulated saliva was collected by passive drooling for 5 ml into a sterile container. After collection, samples were immediately placed on dry ice and subsequently moved to long-term storage at −80 °C within a 4-h window using specialised isothermal transport containers.

### 16S rRNA gene sequencing

Total DNA was extracted from the thawed saliva samples using QIAamp® DNA Microbiome Kit (QIAGEN, Hilden, Germany) following the manufacturer's instructions. The V3-V4 hypervariable region of 16S ribosomal RNA (rRNA) gene was amplified using primers Bakt_341F (5′-CCTACGGGNGGCWGCAG-3′) and Bakt_805R (5′-GACTACHVGGGTATCTAATCC-3′). Sequencing libraries were then constructed from the 16S amplicons using Nextera XT Index Kit and sequenced on the Illumina MiSeq platform with paired-end 300 bp sequences.

### Sequence processing and taxonomic assignment

Read filtering and denoising were performed through the DADA2 pipeline implemented in QIIME 2 version 2024.2 [17]. We used a naïve Bayes classifier from the QIIME 2 plugin for assigning taxonomy to each amplicon sequence variant (ASV). The eHOMD 16S rRNA RefSeq (version 15.23) from Human Oral Microbiome Database (http://www.ehomd.org) was used as a reference for training. After taxonomic assignment through the classify-sklearn function, any ASVs having a confidence value <0.7 were marked as ‘unassigned’. Due to the limited length of the V3-V4 region, many ASVs can be assigned to a genus level but not a species level. Therefore, we performed downstream analyses in parallel for both genus and species levels to identify bacterial taxa that are associated with caries status (Figure S1).

For a feature table from DADA2 denoising, we performed additional data filtering as follows. Any ASVs that have less than 10 read counts in any samples were removed. Rarefaction analysis was performed to normalise sequencing depth through the vegan package (version 2.7-2) performed in R.

### Data analyses

All statistical computations and visualisations were performed using the R statistical environment (version 4.5.2) and RStudio (version 2025.09.2).

#### Demographic and clinical data analysis

Statistical differences in demographic and clinical variables, including age, duration of imprisonment, number of remaining teeth, decayed teeth (DT) and filled teeth, were evaluated between the CA and CF groups. Due to the non-normal distribution of these variables (confirmed via the Shapiro‒Wilk test), the Mann‒Whitney U test was employed. The association between caries status and other health conditions (controlled hypertension, controlled diabetes, history of smoking) was assessed using the Fisher's exact test.

#### Microbiota diversity analysis

The filtered ASV feature table was aggregated to the class, genus and species taxonomic levels. ASVs assigned to the same taxon were merged, and read counts were summed. Unclassified ASVs were labelled as ‘Unassigned’ or ‘Genus unassigned’ where appropriate. Stacked bar plots representing class and genus compositions were generated using the ggplot2 R package (version 4.0.1).

Microbial diversity analyses were performed using the vegan package in R. To account for the sensitivity of alpha diversity indices to varying library sizes, samples were rarefied to a uniform depth of 5,500 reads (Figure S2) exclusively for alpha diversity analysis, which included the observed richness, Shannon‒Wiener and Simpson indices. For all subsequent analyses, including beta diversity, differential abundance testing and correlations with caries severity, unrarefied taxa counts were normalised to relative abundances to preserve the full depth of the dataset. Beta diversity was characterised using Bray–Curtis dissimilarity matrices and visualised via Non-Metric Multidimensional Scaling (NMDS). Statistical significance in community structure (centroids) between the caries-active (CA) and caries-free (CF) groups was evaluated using Permutational Multivariate Analysis of Variance (PERMANOVA). Additionally, multivariate homogeneity of group dispersions (biological variations) was assessed using the betadisper function followed by the Tukey's honest significant difference (HSD) test. To evaluate the impact of potential confounding variables, these diversity analyses were replicated across groups stratified by hypertension status and smoking history.

#### Identification of caries-associated taxa

Bacterial taxa associated with caries status were evaluated based on both presence–absence patterns and differential abundance. To reduce multiple comparison bias, taxa present in less than 25% of samples were filtered out, resulting in a final dataset of 62 genera and 110 species.

‐ Presence–absence: indicator species analysis was performed using the indicspecies package (version 1.8.0) to identify taxa predominantly associated with either the CA or CF group.
‐ Differential abundance: differences in relative abundance between groups were tested using the Mann‒Whitney U test, accompanied by Cliff's delta (effect size) and log_2_-fold change calculations.

The *p*-values were adjusted for multiple comparisons using the Benjamini‒Hochberg false discovery rate (FDR) method. An adjusted *p*-value < 0.25 was considered statistically significant, a threshold previously suggested for exploratory microbiome studies [18]. A similar workflow was applied to screen for taxa associated with hypertension and taxa associated with smoking history.

#### Correlation with caries severity

To investigate the relationship between the microbiota and caries severity, the ‘percentage of teeth with active caries’ (number of decayed teeth/total remaining teeth × 100) was defined as a severity proxy. This analysis was restricted to the caries-active group (*n* = 14). Taxa present in less than 50% of these subjects (<7 samples) were excluded, leaving 51 genera and 67 species.

The Spearman's rank correlation test was performed to examine associations between caries severity and taxonomic relative abundance. After FDR correction, an adjusted *p-*value < 0.25 was used as the significance cutoff. To control for potential confounding factors, a comparable approach was employed to assess the correlations between taxonomic relative abundance and subject age, duration of imprisonment and the number of remaining teeth. Finally, all results from differential abundance and correlation analyses were visualised using volcano plots, heatmaps and bar plots generated via the vegan, ggplot2 and pheatmap (version 1.0.13) packages.

## Results

### Demographic and clinical characteristics

The demographic and clinical characteristics of the study population are summarised in Table 1. The study cohort consisted of 28 participants with a mean age of 62.9 ± 7.7 years and a mean duration of imprisonment of 8.0 ± 5.2 years.

**Table 1.** Demographic data of subjects included in this study.

Properties	All (*n* = 28)	Caries-active group (*n* = 14)	Caries-free group (*n* = 14)	*p*-value for the difference between groups^a^
Sex	All male	All male	All male	
Age	62.9 ± 7.7	63.9 ± 7.5	61.9 ± 8.0	0.595^a^
Years of imprisonment	8.0 ± 5.2	7.8 ± 3.5	8.2 ± 6.5	0.796^a^
Total remaining teeth	24.7 ± 5.6	25.1 ± 6.6	24.3 ± 4.7	0.564^a^
Total caries teeth	NA	5.1 ± 4.3	0	**<**0.050^*^^a^
Total filled teeth	8.4 ± 5.8	7.5 ± 6.6	9.1 ± 5.0	0.342^a^
Number of subjects with controlled hypertension	12	6	6	0.648^b^
Number of subjects with controlled diabetes^c^	8	5	3	0.339^b^
Number of subjects with smoking history^d^	11	6	5	0.500^b^

^a^ The Mann‒Whitney U test for age, years of imprisonment, total remaining teeth, total caries teeth and total filled teeth.

^b^ Fisher's exact test for the association between controlled hypertension, controlled diabetes or smoking history and caries status.

^c^ All subjects with controlled diabetes also have controlled hypertension.

^d^ All subjects are currently non-smokers. Subjects with a smoking history have quit smoking for at least 5 years.

^*^ Statistically significant (*p*-value < 0.05); total caries teeth as a variable for grouping: Caries-Active (D2T > = 2) and Caries-Free (D2T = 0).

Statistical analyses revealed no significant differences between the CA and CF groups regarding age, years of imprisonment, number of remaining teeth or number of filled teeth (*p* > 0.05). Furthermore, Fisher's exact tests indicated no significant association between caries status and systemic conditions, specifically hypertension, diabetes and smoking history.

### Sequencing, ASV calling and taxonomic assignment

There were 3,271,463 raw pair-ended reads from 16S sequencing of the 28 samples. After read filtering and denoising through the DADA2 pipeline, there were 338,298 reads from all 28 samples present in a feature table with 5,000 amplicon sequence variants (ASVs). Among these, 1,478 ASVs can be assigned to a genus level but not a species level (Table S2–S3). After additional filtering of low-abundance ASVs (less than 10 read counts in any samples), 2,424 ASVs remained in a feature table. To account for the variation in sequencing depth across the 28 samples, which ranged from 5,514 to 21,636 reads (Figure S2A), we performed rarefaction to normalise library sizes. Consequently, all samples were subsampled to a uniform depth of 5,500 reads per sample for alpha diversity analyses (Figure S2B).

### Bacterial composition of the salivary microbiota

**Figure 1.**
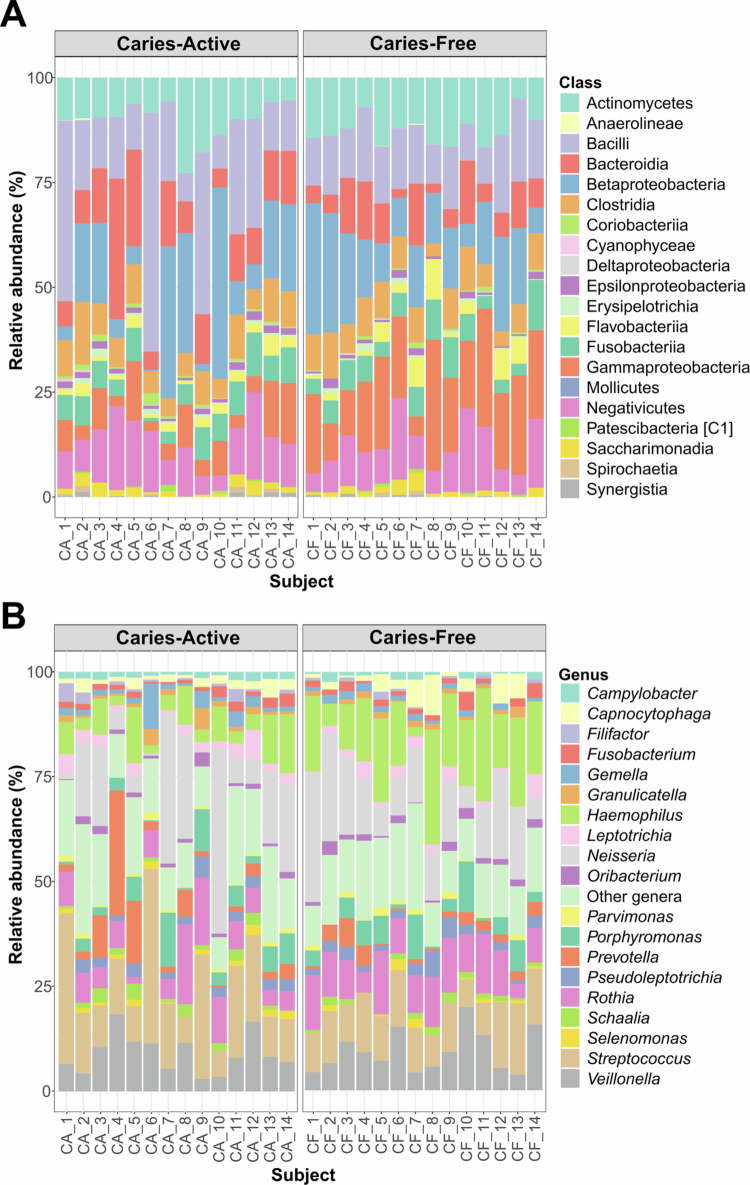
Compositional plots showing bacterial diversity for Class (A) and Genus (B) levels. For panel (B), only the top 20 genera with the highest mean of %abundance were displayed in, and the rest of genera were combined and assigned as ‘Other genera’.

The 2,424 ASVs from the filtered feature table were classified into 20 classes, 122 genera and 303 species (Figure 1). At the class level, the CA group was dominated by Bacilli (21.7%), Betaproteobacteria (15.4%), Bacteroidia (12.3%), Negativicutes (11.2%) and Actinomycetes (10.4%). In contrast, the top five most abundant classes in the CF group were Gammaproteobacteria (18.2%), Betaproteobacteria (16.0%), Bacilli (13.8%), Actinomycetes (12.6%) and Negativicutes (10.4%) (Figure 1A).

At the genus level, *Haemophilus*, *Neisseria*, *Streptococcus*, *Rothia* and *Veillonella* were the five most abundant genera across both groups. Notably, the relative abundance hierarchy differed: *Streptococcus* was the most predominant genus in the CA group (17.3%), whereas *Haemophilus* was the most abundant in the CF group (15.9%) (Figure 1B).

Regarding species composition, the CA group was characterised by high mean abundances of *Rothia mucilaginosa* (10.9%), *Haemophilus parainfluenzae* (10.2%), *Prevotella melaninogenica* (6.3%), *Porphyromonas pasteri* (5.7%) and *Veillonella rogosae* (5.3%). Conversely, the CF group exhibited a profile dominated by *H. parainfluenzae* (22.1%), followed by *R. mucilaginosa* (13.0%), *V. rogosae* (6.7%), *P. pasteri* (4.9%) and *Veillonella tobetsuensis* (2.98%).

### Diversity analyses of salivary microbiota profiles

#### Caries status analysis

**Figure 2.**
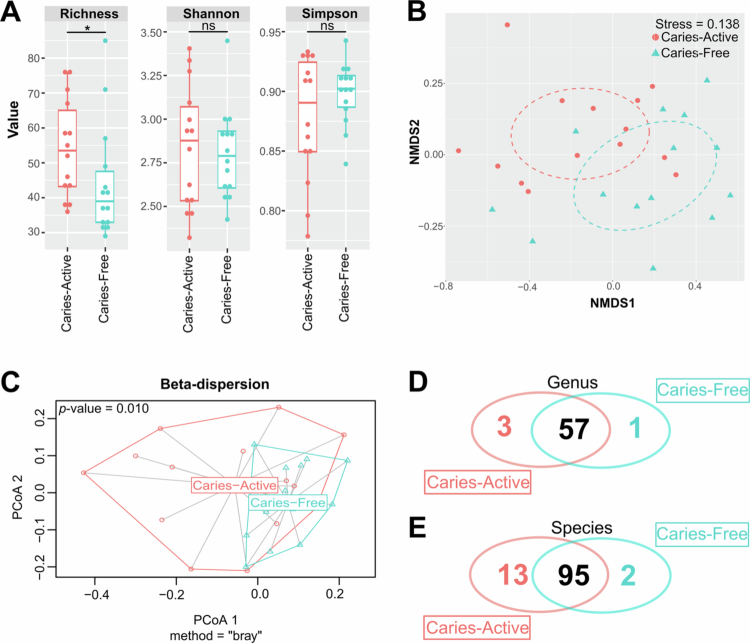
Genus-level diversity analysis (A) Alpha diversity showing richness, Shannon‒Weiner diversity and Simpson diversity between caries-active (CA) and caries-free (CF) groups. Note that only richness is significantly (*p*-value < 0.05). (B) Beta diversity showing ordination of microbiome profiles between two groups. (C) Beta dispersion plot depicting the different variability of bacterial profiles between the CA and CF groups. (D and E) Venn diagram depicting a number of bacterial genera (D) and species (E) specific to certain groups, as indicated by the multi-pattern analysis (Tables S4 and S5).

Alpha diversity analysis at the genus level revealed that Observed Richness was significantly higher in the CA group compared to the CF group (Mann–Whitney U = 145.5, *p* = 0.031, Cliff's *δ* = 0.485). While no significant differences were observed for the Shannon–Wiener and Simpson indices, the CA group exhibited greater inter-individual variation in these metrics compared to the CF group (Figure 2A).

Regarding beta diversity, the NMDS plot displayed distinct clustering between the CA and CF groups (Figure 2B). PERMANOVA analysis confirmed a significant difference in microbial community structure between the two groups (*R*² = 0.114, *p* = 0.002). Furthermore, analysis of multivariate homogeneity of group dispersions (PERMDISP) revealed a significant difference (*p* = 0.010; Figure 2C), indicating that the CA group possessed significantly higher microbiota heterogeneity (beta-dispersion) compared to the CF group.

#### Confounding factor analysis: hypertension and smoking history

To assess potential confounding effects, we compared microbiota profiles between subjects stratified by hypertension status (controlled vs. absent) and smoking history (former smokers vs. never-smokers). No significant differences in alpha diversity metrics (Richness, Shannon and Simpson indices) were observed between subjects stratified by hypertension status (Figure S3A). Similarly, beta diversity and beta-dispersion analyses showed no distinct separation (Figure S3B and C). PERMANOVA results confirmed that the bacterial communities did not differ significantly based on hypertension status (*R*² = 0.021, *p* = 0.846). Similarly, no significant differences were observed in alpha diversity indices (Figure S4A), microbial community composition (PERMANOVA *R*^2^ = 0.032, *p* = 0.804, Figure S4B) and beta dispersion (Figure S4C) when comparing subjects based on smoking history.

### Bacterial taxa associated with the subject group

#### Taxa associated with caries status

Indicator species analysis (presence–absence patterns) identified specific taxa associated with caries status. At the genus level, *Megasphaera*, Paludibacteraceae [G-1] and Anaerovoraceae [G2] were significantly associated with the CA group, while *Riemerella* was specific to the CF group (Figure 2D, Table S4). At the species level, two taxa were specific to the CF group: *Aggregatibacter* sp. HMT-949 and *Riemerella* sp. HMT-432. In contrast, 13 species were significantly associated with the CA group, including *Dialister invisus*, *Megasphaera micronuciformis*, *Prevotella intermedia*, *Selenomonas sputigena* and *Capnocytophaga ochracea* (Figure 2E, Table S5).

**Figure 3.**
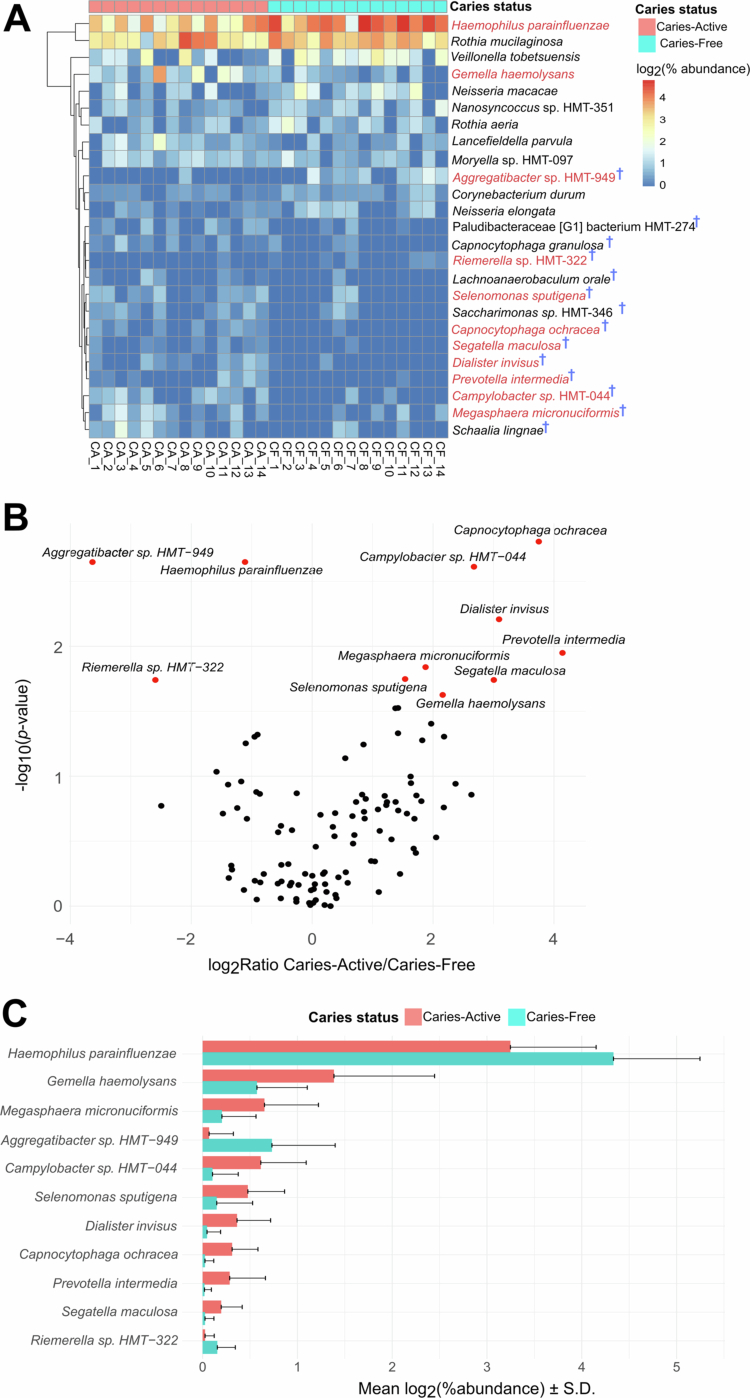
Abundance of bacterial composition at the species level (A) Heatmap showing abundance of bacterial species in all 28 subjects. Only the top 20 species with the highest absolute value of Cliff's delta were shown in the heatmap. Red texts indicate species that are significantly different between CA and CF groups from the Mann–Whitney U test (Table S7). A dagger next to a tip text indicates species that are predominantly found in a certain group based on multi-pattern analysis (Table S5). (B) Volcano plot depicting bacterial species that have different abundance between CA and CF groups. Red dots indicated species with significantly abundant (Mann–Whitney U test; multiple testing-adjusted *p*-value < 0.25 (Table S7). (C) Barplots showing abundance values of significant species from the volcano plot.

Differential abundance analysis (Mann‒Whitney U test) further revealed significant quantitative differences. At the genus level, *Haemophilus* and *Aggregatibacter* were significantly different between the CA and CF groups (Figure S4, Table S6). At the species level, 11 taxa exhibited significant differences in relative abundance (Figure 3, Table S7). While most of these overlapped with the taxa identified in the presence/absence analysis (Table S5), three additional species were notably enriched: *Campylobacter* sp. HMT-044 (log_2_ ratio = 2.676) and *Gemella haemolysans* (log_2_ ratio = 2.159) in the CA group, and *Haemophilus parainfluenzae* (log_2_ ratio = −1.248) in CF group.

#### Taxa associated with hypertension and smoking history (confounding analysis)

Regarding the potential influence of hypertension, indicator analysis identified two genera and six species associated with the controlled hypertension group, including *Riemerella*, Anaerovoracaceae [G-2], *Leptotrichia wadei*, *Treponema denticola*, *Prevotella intermedia* and *Peptoanaerobacter yurii* (Figure S3D and S3E, Tables S8–S9). However, differential abundance analysis showed no statistically significant differences in the relative abundance of any genus or species between subjects with and without controlled hypertension (Figure S5, Tables S10–S11).

For testing among groups with/without smoking history, none of the genus/species was neither associated nor enriched in any groups (Figure S4D and E, Tables S12–S15). Collectively, the results from both global diversity and individual taxonomic analyses suggest that controlled hypertension and smoking history (currently non-smokers) had a negligible effect on the salivary bacterial composition in this cohort. Our result in the smoking history also implied the restoration of oral microbiota after quitting cigarette smoking for years, as seen in a previous large-scale study in American populations [19].

### Correlation between caries severity and bacterial abundance

**Figure 4.**
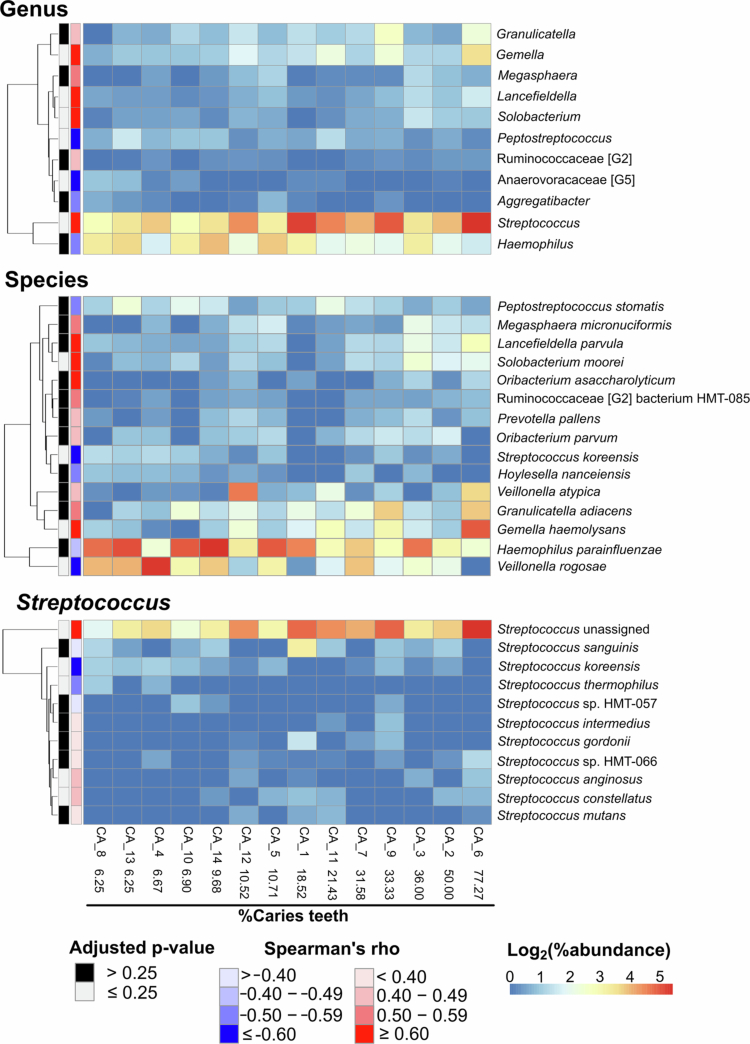
Heatmaps showing correlation between %caries teeth (horizontal axis) and %abundance of bacterial genus and species, as well as species of *Streptococcus* (cell colour). For the first two panels, only taxa with Spearman's rho values ≥0.40 or ≤−0.40 are present in the heatmaps. For the *Streptococcus* panel, all assigned species are reported. Multiple comparison corrections were performed separately for the species (*n* = 67) and Streptococcus (*n* = 11) panels to reflect the total number of taxa tested in each group. Note that most ASVs of *Streptococcus* cannot be taxonomically assigned to the species level (Table S3). Cell colours display log_2_-transformed relative abundance. The sidebar indicates Spearman's rho values (blue = negative, red = positive) and significance (adjusted *p*-value > or ≤ 0.25). Taxonomic rows were clustered using Euclidean distance metrics. Detailed correlation results are available in Tables S16–S18.

Spearman's rank correlation analysis was performed to identify bacterial taxa associated with caries severity, using the percentage of active caries teeth as a proxy (Figure 4, Tables S16—S17). At the genus level, 11 genera exhibited moderate-to-strong correlations (Spearman's rho ≥ 0.40 or ≤−0.40). However, only 6 genera achieved statistical significance. *Gemella* (rho = 0.748), *Solobacterium* (rho = 0.682), *Lancefieldella* (rho = 0.645) and *Streptococcus* (rho = 0.627) showed significant positive correlations with caries severity, while *Peptostreptococcus* (rho = −0.640) and *Anaerovoracaceae* [G-5] (rho = −0.606) showed a significant negative correlation (Table S16).

At the species level, 4 species displayed strong, statistically significant correlations (absolute rho > 0.60). *Gemella haemolysans* (rho = 0.658) and *Solobacterium moorei* (rho = 0.640) were positively correlated with caries severity, while *Veillonella rogosae* (rho = −0.669) and *Streptococcus koreensis* (rho = −0.678) were negatively correlated. Additionally, taxa previously identified as differentially abundant between the CA and CF groups also correlated with severity, including *Megasphaera micronuciformis* (rho = 0.555) and *Haemophilus parainfluenzae* (rho = −0.493) (Table S17).

#### *Streptococcus* species analysis

Given the strong positive correlation observed between the genus *Streptococcus* and caries severity, a detailed species-level analysis was conducted (Figure 4, Table S18). A total of 10 *Streptococcus* species were detected, including members of the *Streptococcus mutans* group. However, significant correlations were observed for only four species: *S. koreensis* (rho = −0.678) and *Streptococcus thermophilus* (rho = −0.536) showed negative correlations, while *Streptococcus anginosus* (rho = 0.485) and *Streptococcus constellatus* (rho = 0.463) showed a positive correlation (Table S18).

Notably, these identified species represented a relatively low proportion of the total *Streptococcus* abundance (Figure 4). The majority of ASVs assigned to this genus could not be classified to the species level (Table S3). This limitation is likely attributed to the insufficient taxonomic resolution of the V3–V4 amplicon region for differentiating closely related *Streptococcus* species.

#### Analysis of potential confounders

To evaluate the impact of potential confounding variables on microbial composition, Spearman's rank correlation analyses were performed to assess associations between taxonomic relative abundance and subject age, duration of imprisonment and the number of remaining teeth (Tables S19–S24).

Regarding subject age, the relative abundances of five genera and five species were significantly correlated with age (Tables S19–S20). Specifically, *Veillonella rogosae* (rho = −0.692) and *S. koreensis* (rho = −0.693) were the only two species negatively associated with age. Notably, these two taxa were also negatively associated with caries severity; however, no other age-associated taxa overlapped with those identified as caries-associated signatures (Tables S16–S20). Similarly, while the abundances of 15 genera and five species were significantly associated with the duration of imprisonment, none of these taxa overlapped with those identified in the caries severity analysis (Tables S21–S22).

Finally, there were 6 genera and 12 species that had relative abundance correlated with the number of remaining teeth. Many of these taxa overlapped with caries-associated taxa (Tables S16—S18, S23—S24), including *Peptostreptococcus* (rho 0.690), Anaerovoraceae [G5] (rho 0.666), *V. rogosae* (rho = 0.615), *S. koreensis* (rho = 0.560), *G. haemolysans* (rho = −0.644), *S. moorei* (rho = −0.587) and *Streptococcus* (rho = −0.644). It was notable that the number of remaining teeth in CA subjects has a strong negative correlation to caries severity (rho = 0.781, *p-*value < 0.001), which is consistent with a previous study that tooth loss by extraction is a common intervention for dental caries in incarcerated prisoners [20]. However, subject age (rho = 0.510, *p-*value 0.062) and subject year of imprisonment (rho = −0.036, *p*-value 0.913) did not have a significant correlation with caries severity in this cohort.

## Discussion

Epidemiological surveillance has long established that incarcerated populations suffer disproportionately from oral diseases, particularly dental caries and periodontitis [14,21,22]. This disparity is most acute among older inmates, who exhibit significantly higher caries [23]. While the structural and behavioural determinants of poor oral health in this setting are well understood, the underlying microbial ecology has been largely overlooked. To date, there is a paucity of data regarding the specific bacterial taxa associated with caries in this demography.

This study provides a novel characterisation of the salivary microbiota in older incarcerated adults, bridging the gap between clinical observation and microbiological aetiology. A fundamental strength of this investigation is the carceral setting; its inherent uniformity, characterised by standardised nutrition and systematised routines, mitigates confounding factors such as lifestyle heterogeneity and variable oral hygiene compliance. By minimising the environmental noise typical for free-living populations, this study facilitates a high-resolution analysis of the associations between the oral microbiome and dental caries severity.

Regarding alpha diversity, our analysis identified that species richness was significantly elevated in the CA group, similar to a few previous studies [24,25]. This finding diverges from the majority of existing salivary and plaque microbiome literature, which has predominantly reported either no significant difference [9,16,26–28] or reduced diversity associated with caries status [29,30]. In terms of community structure, beta diversity analysis, specifically beta dispersion, revealed significantly greater compositional variability within the CA group compared to the CF controls. While beta dispersion is frequently underreported, our findings align with several studies that indicate a wider divergence of microbial profiles in disease states. For instance, Yang et al. [31] observed similar heterogeneity in the salivary microbiomes of young adults, and Wolff et al. [30] reported markedly higher variability in carious dentin compared to healthy surfaces. Analogous trends have been observed in plaque microbiomes, where healthy subjects exhibit tighter clustering compared to those with early childhood caries [32], a pattern also noted, though non-significantly, by Jiang et al. [27]. Collectively, these data support the hypothesis that the healthy oral microbiome is maintained by strict homoeostatic constraints [33,34]. The onset of caries appears to disrupt this conservation, leading to dysbiosis characterised by increased stochasticity and compositional heterogeneity.

To contextualise our results, we cross-referenced the differentially abundant taxa in our cohort with established microbiome profiles from previous studies (Figure 3 and S5; Tables S4—S7, S25). Our analysis identified *Haemophilus* (specifically *H. parainfluenzae*) as a key biomarker of the CF state, a finding consistent with multiple independent investigations [9,16,29–31,35]. Additionally, we identified *Aggregatibacter* (*Aggregatibacter* sp. HMT-949) and *Riemerella* sp. HMT-322 as significant indicator of a CF oral environment; to the best of our knowledge, these taxa have not been previously linked to caries-free status.

The association of these taxa with oral health is likely driven by specific metabolic and competitive advantages. *H. parainfluenzae* plays a critical role in pH homoeostasis through the reduction of nitrate and nitrite to ammonia. This metabolic activity buffers the oral environment, neutralising acidity and suppressing the growth of acidophilic competitors such as *Streptococcus mitis, S. australis and S. sanguinis* [36].

Importantly, our study observed a distinct inverse correlation between the abundance of these taxa and clinical disease markers: as caries severity increased, the relative abundance of *H. parainfluenzae* declined (Figure 4; Tables S17). This depletion pattern reinforces the hypothesis that these organisms function as protective commensals, and their loss is a precursor to, or consequence of, the shift toward a cariogenic environment.

Analysis of the CA microbiota revealed a dominance of taxa frequently associated with anaerobic and proteolytic environments. Consistent with prior literature, we observed significant elevations of *Megasphaera micronuciformis*, *Dialister invisus* and *Capnocytophaga* in the disease group [9,26,35,37]. Notably, *Dialister invisus*, an asaccharolytic anaerobe, was highly prevalent. This taxon is historically linked to endodontic infections and periodontal pockets [38] and has been identified in the necrotic pulp of severe early childhood caries (S-ECC) [39], suggesting that the cariogenic environment in this population supports a highly anaerobic community.

While the genus *Prevotella* is a well-established marker of caries activity [16,28,29,35], our study specifically identified *Prevotella intermedia* as a significant indicator taxon. Although *P. intermedia* is less frequently reported in standard caries studies compared to other *Prevotella* species, its presence here may reflect the unique clinical profile of this older, incarcerated cohort. Furthermore, we identified several taxa typically associated with periodontitis and aggressive oral disease, including *Selenomonas* (specifically *S. sputigenea*) and *Solobacterium moorei*. *Selenomonas* has been observed in S-ECC cases characterised by low *S. mutans* abundance [40], while *S. sputigenea* and *S. moorei* are established pathobionts in aggressive periodontitis, endodontic infection and halitosis [41,42]. Collectively, the enrichment of these taxa indicates that the ‘caries-active’ microbiota in older inmates is a complex, polymicrobial entity that overlaps significantly with periodontal and necrotic disease profiles, expanding the traditional understanding of cariogenesis in vulnerable adult populations.

The roles of several taxa in dental caries remain a subject of debate in the literature. For instance, while some studies reported a higher salivary abundance of *Gemella* in caries-active (CA) children compared to controls [43,44], others identified *G. haemolysans* as more prevalent in healthy oral cavities [45,46]. Furthermore, Miyoshi et al. demonstrated that *G. haemolysans* culture supernatants can inhibit the growth of *Porphyromonas gingivalis*, a recognised periodontal pathogen. In the present study, *G. haemolysans* was not only enriched in the CA group [46] (Table S7) but also showed a strong positive correlation with caries severity (Tables S16–S17). Collectively, these findings suggest that *G. haemolysans* may function as an opportunistic member of a dysbiotic community or serve as a marker for the transition from health to a cariogenic state.

*Veillonella* represents another genus with a potentially multifaceted role in cariogenesis. As biofilm-forming anaerobes, these bacteria can metabolise lactate and produce nitrite, which may help stabilise oral pH [47,48]. Conversely, research has shown that *Veillonella tobetsuensis* can facilitate *Streptococcus* proliferation and biofilm formation through signalling molecules [49]. In our study, *V. rogosae* was the only taxon whose relative abundance negatively correlated with caries severity (Table S17), a finding consistent with its proposed protective role. However, *V. rogosae* also correlated with confounding variables, including participant age and the number of remaining teeth (Tables S20, S24). Moreover, the difference in *V. rogosae* abundance between the CA and CF groups was negligible and statistically non-significant (log_2_FC = −0.334, adjusted *p*-value = 0.492). Consequently, the functional contribution of *Veillonella* to cariogenesis must be interpreted with caution. Within this incarcerated cohort, cariogenesis may reflect a broad ecological shift in the oral microbiome, characterised by the adaptation of commensal taxa to acidic environments, rather than the presence or absence of a single pathogen. These findings underscore the polymicrobial nature of dental caries and highlight the importance of viewing the disease through the lens of community-wide dysbiosis.

Given the established role of the *mutans* streptococci and *Lactobacillus* species in cariogenesis [7], we assessed their abundance relative to disease status. Consistent with the literature, the genus *Streptococcus* showed a positive correlation with caries severity, exhibiting significant enrichment in the CA group (Figures 3–4, Table S18). This aligns with previous reports linking elevated streptococcal loads to increased DMFT/DMFS indices [47,48].

However, the interpretation of our findings is constrained by the modest sample size and the insufficient taxonomic resolution required to isolate specific cariogens. Of the 393 amplicon sequence variants (ASVs) classified as *Streptococcus*, 287 could not be resolved to the species level. *Streptococcus mutans* was identified in only six of 28 subjects, and *Lactobacillus* reads were negligible, resulting in insufficient statistical power for specific analysis (Tables S2–S3). The infrequent detection of *S. mutans* likely reflects its niche-specific nature as a biofilm-dependent organism, which is often underrepresented in saliva compared to dental plaque. Additionally, the V3–V4 16S rRNA gene amplicon provides limited resolution for the genus *Streptococcus*. As previously reported by Yu et al. [49], the 16S rRNA gene is frequently inadequate for resolving closely related streptococci, suggesting that high-resolution markers like the 16S–23S ITS region are more suitable for species-level characterisation.

This study has several limitations that warrant consideration for future research aimed at elucidating the oral microbiota of incarcerated populations. As an initial investigation into this vulnerable group, we utilised whole saliva collection due to its non-invasive nature and its capacity to capture a higher breadth of microbial diversity compared to other sampling sites [25,50,51]. While salivary biomarkers are effective for assessing general caries status [25,51], site-specific plaque sampling offers superior resolution for investigating the localised mechanisms of cariogenesis [52,53]. Integrating niche-specific sampling in future studies would mitigate the dilution effects inherent to salivary diagnostics, enabling a more precise characterisation of cariogenic taxa.

Furthermore, this study represents a preliminary characterisation of the oral microbiota within an incarcerated Thai cohort. Subsequent multi-ethnic and cross-regional investigations are essential to determine whether caries-associated microbial signatures remain consistent across diverse demographic origins and cultural backgrounds. Lastly, although no significant associations were observed between microbial composition and hypertension or smoking history in this cohort, the impact of other systemic comorbidities, such as diabetes mellitus and dyslipidemia, merits further exploration. Larger-scale longitudinal studies are required to fully understand how these systemic factors influence cariogenesis and the structural dynamics of the oral bacterial community.

## Conclusions

**Figure 5.**
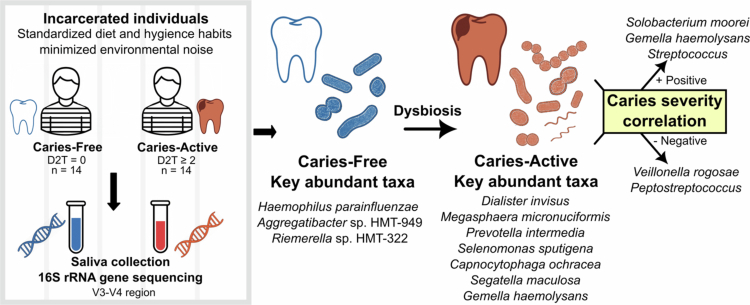
Summary of findings from the salivary microbiome study in older incarcerated adults. The schematic illustrates the study design, comparison between caries-active and caries-free groups, key bacterial taxa associated with each group and microbial signatures correlated with caries severity.

In summary (Figure 5), this study elucidates distinct salivary microbiota profiles associated with caries status in older incarcerated individuals. Our data suggest that the caries-free state is characterised by ecological stability and the conservation of health-associated commensals, specifically *H. parainfluenzae*. Conversely, the caries-active state is defined by a distinct dysbiosis: the depletion of these protective taxa coincides with the proliferation of a diverse consortium of anaerobic and cariogenic pathobionts, including *D. invisus*, *M. micronuciformis*, *C. ochracea*, *P. intermedia*, *S. sputigenea*, *S. moorei* and the genus *Streptococcus*. As a pioneering investigation, this work provides foundational insight into the microbial dynamics of cariogenesis within a controlled carceral setting, offering a baseline for future oral health interventions in this vulnerable population. Ultimately, these findings pave the way for a transition toward precision-guided oral care, where longitudinal validation of these microbial signatures will be essential for developing diagnostic tools and ecological therapies tailored to the unique physiological and environmental needs of institutionalised populations.

## Supplementary Material

Supplementary Material SupplementaryFile1_Figures_R1_22_Apr_2026_07_30_AU.docx

Supplementary Material SupplementaryFile1_Figures_R1_22_Apr_2026_07_30_AU.docx

## Data Availability

Raw Illumina sequencing data generated in this study were deposited in the NCBI database under the BioProject accession PRJNA1398001. Scripts used in the analyses for this study are available under a reasonable request.
