# Supplementary material for: Microbial signatures of dental caries in the incarcerated elderly: a salivary microbiota study in a restricted environment
Source: J Oral Microbiol. 2026 Apr 29;18(1):2662787. doi: 10.1080/20002297.2026.2662787 (PMC13130238; doi:10.1080/20002297.2026.2662787)
Supplement: Supplementary Material — SupplementaryFile1_Figures_R1_22_Apr_2026_07_30_AU.docx [file ZJOM_A_2662787_SM8579.docx]

**Supplementary Figures**

**Microbial Signatures of Dental Caries in the Incarcerated Elderly: A Salivary Microbiota Study in a Restricted Environment**

Teeratas Kijpornyongpan^1^, Annop Krasaesin^1^, Thananya Chongcharoenkit^2^, Khanti Rattanapornsompong^1^, Avirut Truntipakorn^3^, Patita Bhuridej^4^, Nadnudda Rodthongkum^5^, Sung-Dae Cho^6^ and Thantrira Porntaveetus^1,7^*

^1^Center of Excellence in Precision Medicine and Digital Health, FutureDent Digital Center, Department of Physiology, Faculty of Dentistry, Chulalongkorn University, Bangkok, Thailand

^2^Master of Science Program in Geriatric Dentistry and Special Patients Care, Faculty of Dentistry, Chulalongkorn University, Bangkok, Thailand

^3^Bang Kwang Central Prison, Department of Corrections of Thailand, Ministry of Justice, Nonthaburi, Thailand

^4^Department of Community Dentistry, Faculty of Dentistry, Chulalongkorn University, Bangkok, Thailand

^5^Department of Chemistry, Faculty of Science, Chulalongkorn University, Bangkok, Thailand

^6^Department of Oral Pathology, School of Dentistry and Dental Research Institute, Seoul National University, Seoul, Republic of Korea

^7^Clinic of General, Special Care and Geriatric Dentistry, Center for Dental Medicine, University of Zürich, Zürich, Switzerland

***Correspondence to:**

Thantrira Porntaveetus, DDS, Grad Dip, MSc, PhD

Center of Excellence in Precision Medicine and Digital Health, Department of Physiology, Faculty of Dentistry, Chulalongkorn University, Bangkok 10330, Thailand

Tel: 662-218-8695; E-mail: [thantrira.p@chula.ac.th](mailto:thantrira.p@chula.ac.th), thantrira.porntaveetus@zzm.uzh.ch

ORCID: 0000-0003-0145-9801

Short title: Oral Microbiome in Elderly Prisoners


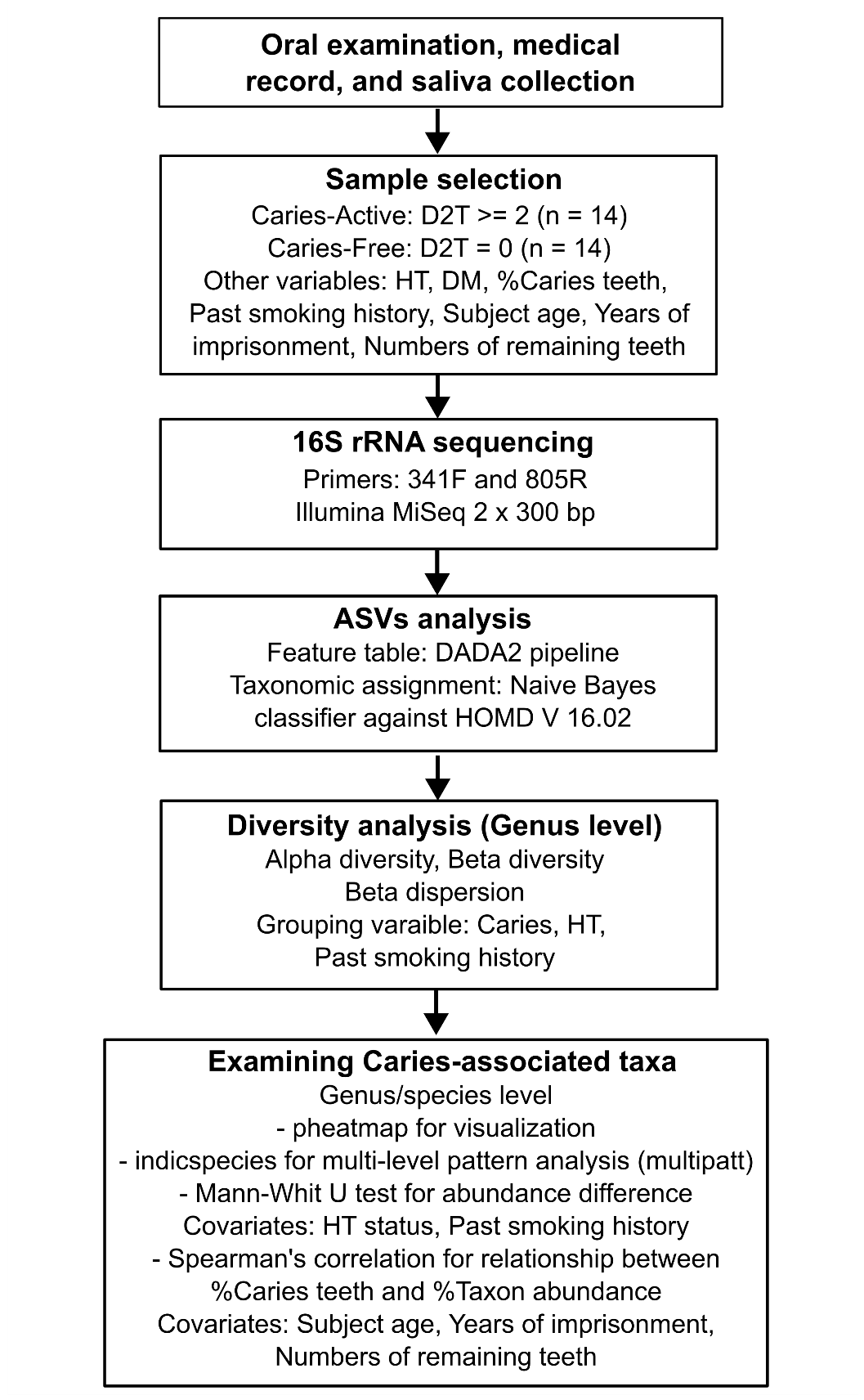


**Figure S1** Analysis pipeline conducted in this study.


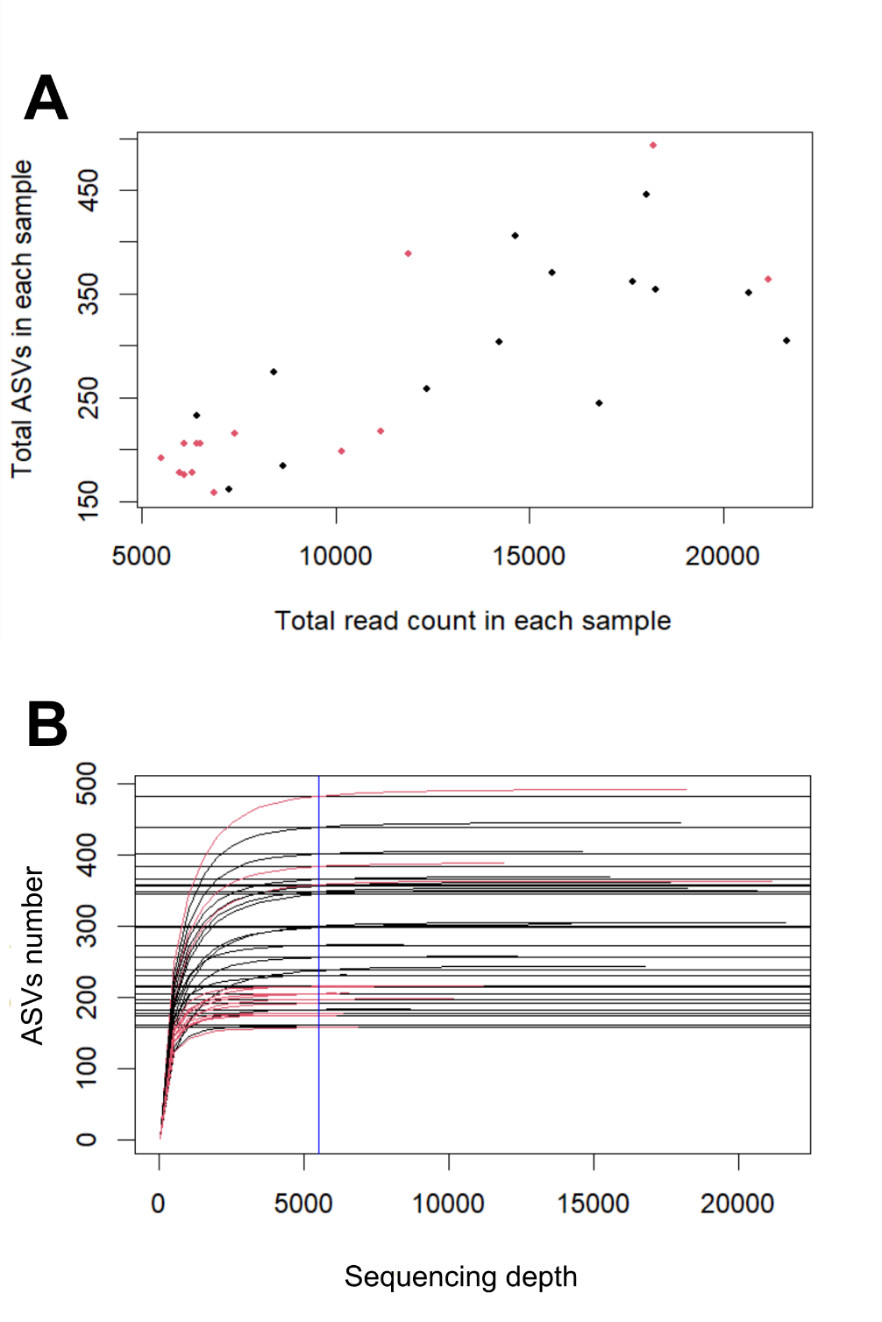


**Figure S2** Rarefaction analysis. (A) Scatterplots showing a relationship between total read count and ASVs richness (B) Rarefaction curve showing cutoff at 5,500 reads for alpha diversity analysis.


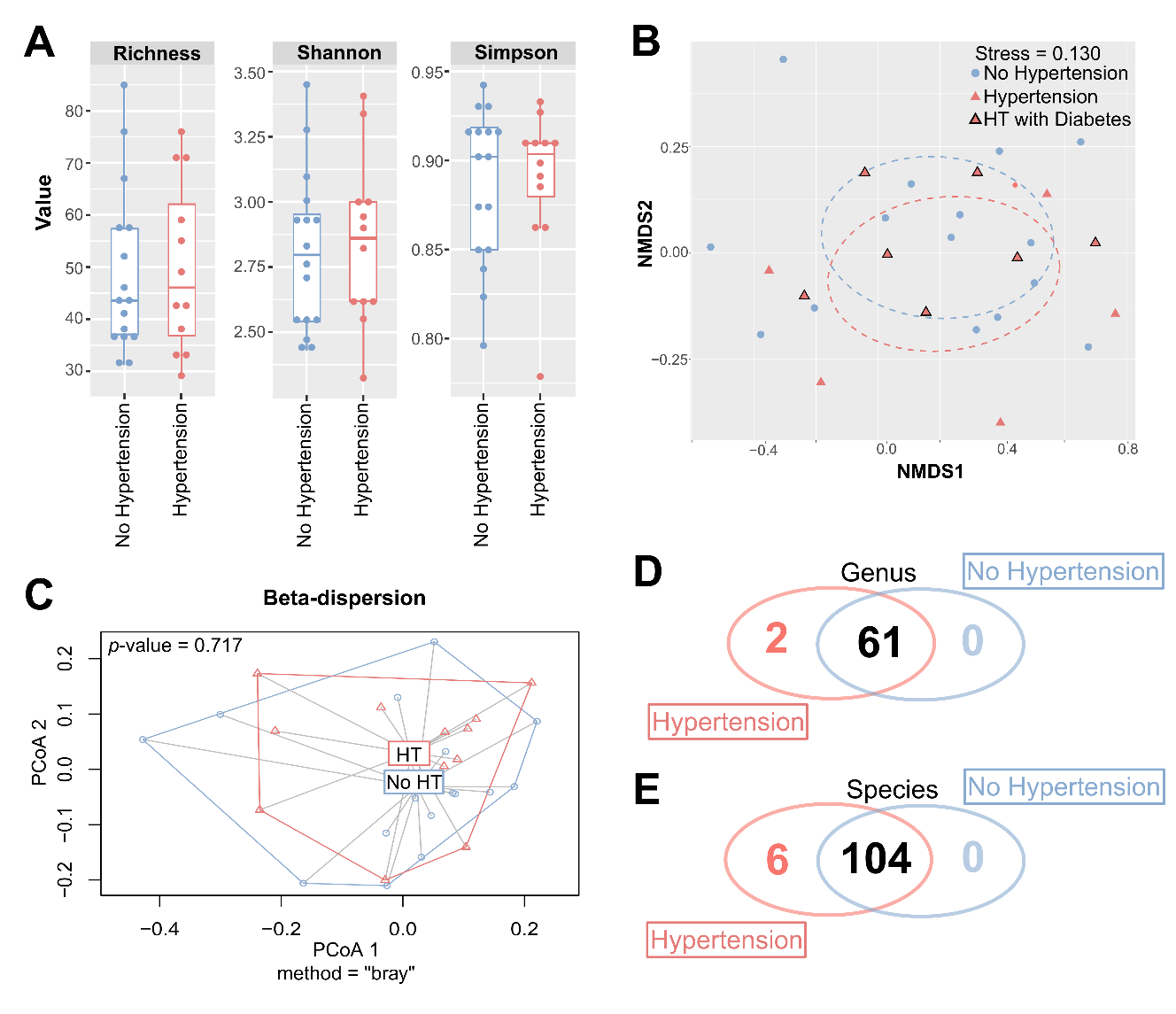


**Figure S3** Genus-level diversity analysis for subject with different hypertension statuses (A) Alpha diversity showing richness, Shannon-Weiner’s diversity, and Simpson diversity between Hypertension and No hypertension groups. None of alpha diversity indices are statistically significant. (B) Beta diversity showing ordination between two groups. (C) Beta dispersion plot depicting different variability of bacterial profiles between two groups. (D and E) Venn-Diagram depicting a number of bacterial genera (D) and species (E) specific to certain groups, as indicated by multi-pattern analysis (Tables S8 and S9).


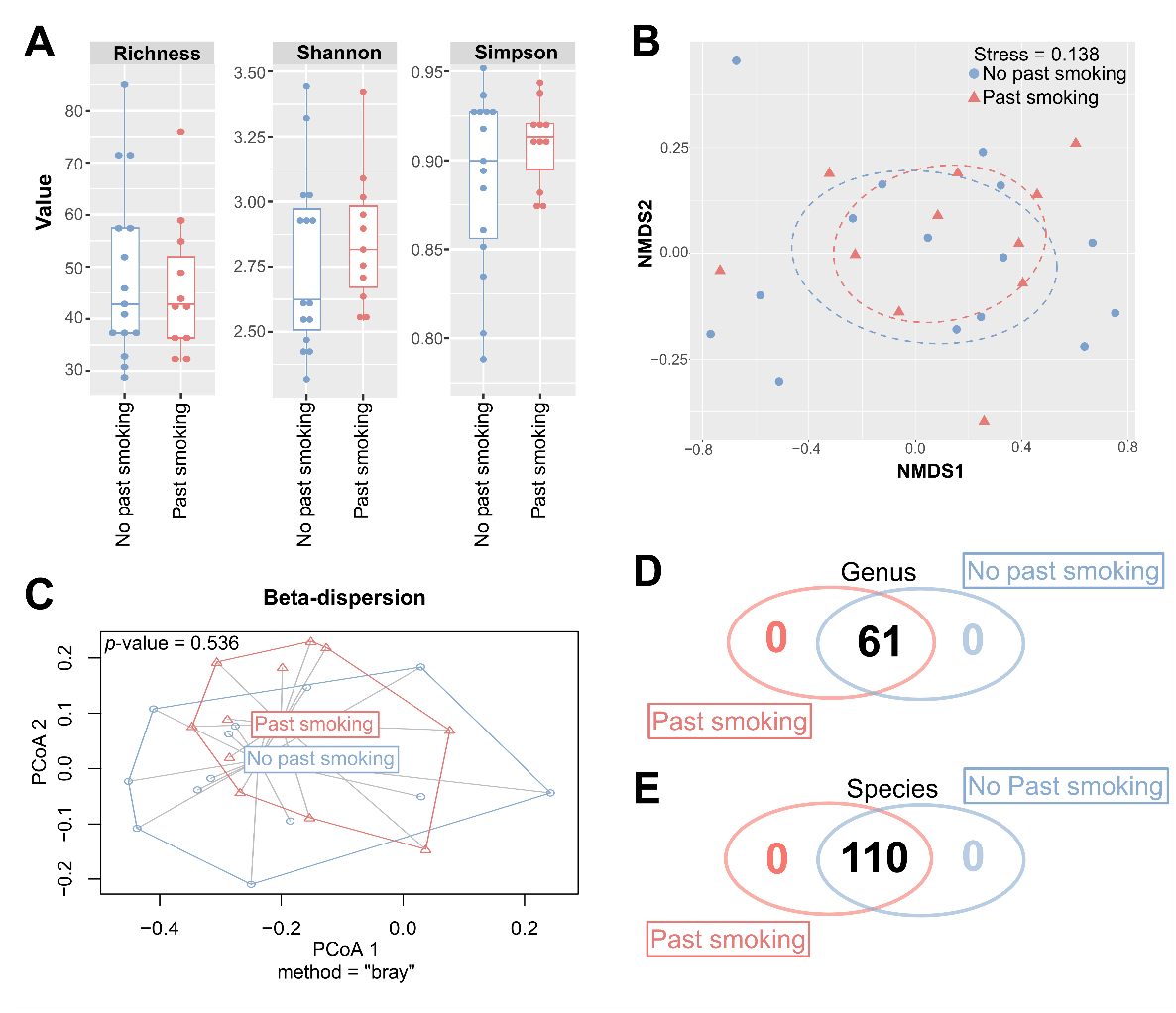


**Figure S4** Genus-level diversity analysis for subjects with different smoking history (A) Alpha diversity showing richness, Shannon-Weiner’s diversity, and Simpson diversity between having smoking history and no smoking history groups. None of alpha diversity indices are statistically significant. (B) Beta diversity showing ordination between two groups. (C) Beta dispersion plot depicting different variability of bacterial profiles between two groups. (D and E) Venn-Diagram depicting a number of bacterial genera (D) and species (E) specific to certain groups, as indicated by multi-pattern analysis (Tables S12 and S13). Note that none of genus and species is specific to any groups classified by smoking history.


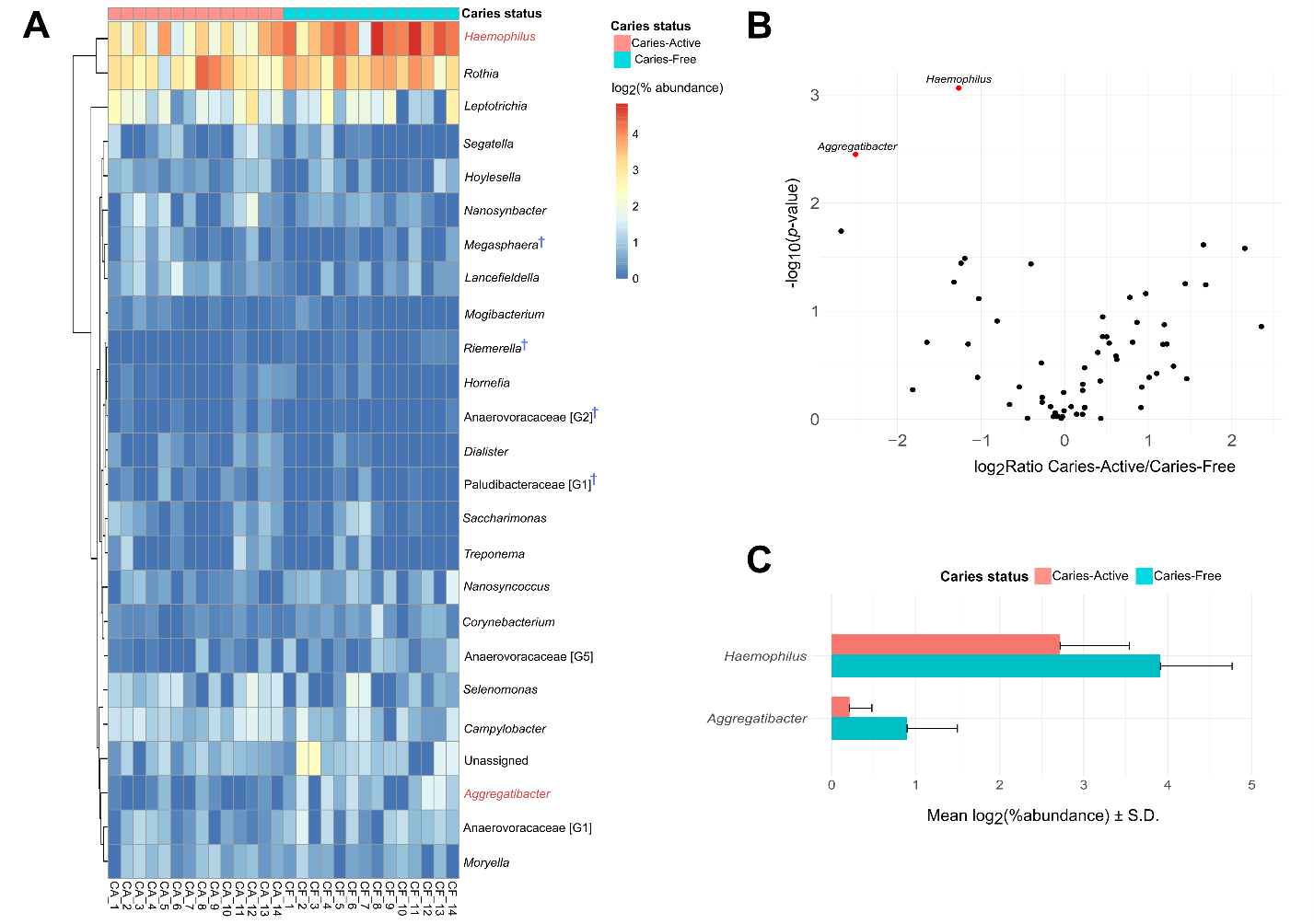


**Figure S5** Abundance of bacterial composition at genus level (A) Heatmap showing abundance of bacterial genera in all 28 subjects. Only top 20 genera with the highest absolute value of Cliff’s delta were shown in the heatmap. Red texts indicate genera that are significantly different between CA and CF groups from Mann-Whitney U test (Table S6). Daggers next to texts indicate genera that are predominantly found in certain group based on multi-pattern analysis (Table S4). (B) Volcano plot depicting bacterial genera that have different abundance between CA and CF groups. Red dots indicated genera with significantly abundant from the Mann-Whitney U test after multiple *p*-value correction with the adjusted *p*-value threshold of 0.25 (Table S6). (C) Barplots showing abundance values of significant genera from the volcano plot.


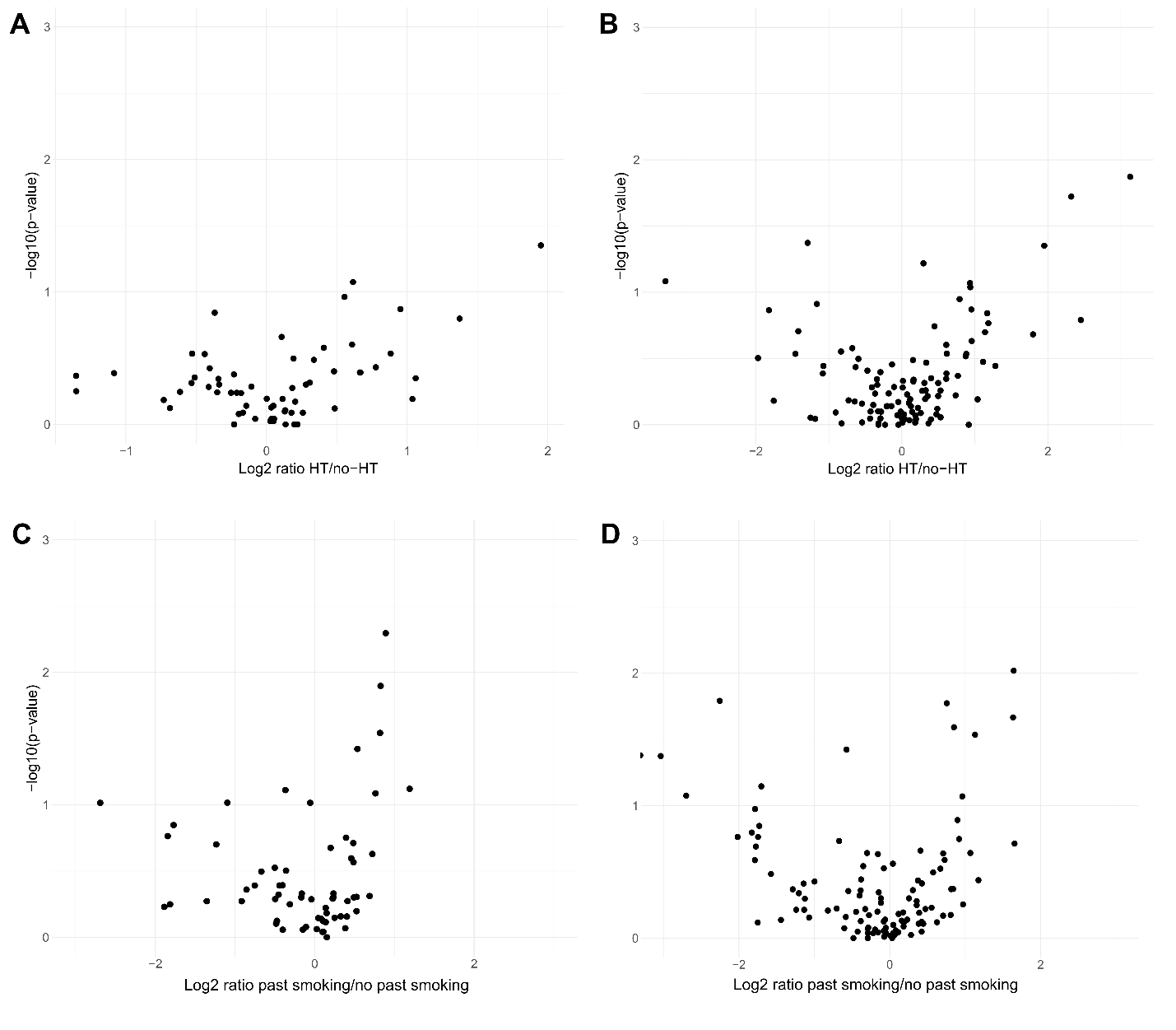


**Figure S6** Volcano plot depicting there is neither bacterial genus (A, C) or species (B, D) that has significantly different abundance between hypertension (HT) and no hypertension (no-HT) (A, B) or significantly different abundance between smoking history and no smoking history (C, D). Note that some taxa have *p*-values from Mann-Whitney U test less than 0.05. However, after *p*-value adjustment, none of these taxa pass the 0.25 cutoff value (Tables S10 – S11, S14 – S15).
